# Oncofetal protein IGF2BPs in human cancer: functions, mechanisms and therapeutic potential

**DOI:** 10.1186/s40364-023-00499-0

**Published:** 2023-06-06

**Authors:** Tian-Yu Zhu, Lian-Lian Hong, Zhi-Qiang Ling

**Affiliations:** 1 Zhejiang Cancer Hospital, Hangzhou, 310022 Zhejiang China; 2 Hangzhou Institute of Medicine (HIM), Chinese Academy of Sciences, Hangzhou, 310018 Zhejiang China; 3 The Second School of Clinical Medicine, Wenzhou Medical University, No.109 Xueyuan West Road, Wenzhou, 325027 Zhejiang China; 4 Jinhua People’s Hospital, No.267 Danxi East Road, Jinhua, 321000 Zhejiang China

**Keywords:** IGF2BPs, m^6^A, Cancer, Therapeutic target

## Abstract

N^6^-methyladenosine (m^6^A) is the most prevalent and well-characterized internal chemical modification in eukaryotic RNA, influencing gene expression and phenotypic changes by controlling RNA fate. Insulin-like growth factor-2 mRNA-binding proteins (IGF2BPs) preferentially function as m^6^A effector proteins, promoting stability and translation of m^6^A-modified RNAs. IGF2BPs, particularly IGF2BP1 and IGF2BP3, are widely recognized as oncofetal proteins predominantly expressed in cancer rather than normal tissues, playing a critical role in tumor initiation and progression. Consequently, IGF2BPs hold potential for clinical applications and serve as a good choice for targeted treatment strategies. In this review, we discuss the functions and mechanisms of IGF2BPs as m^6^A readers and explore the therapeutic potential of targeting IGF2BPs in human cancer.

## Background

**Fig. 1:**
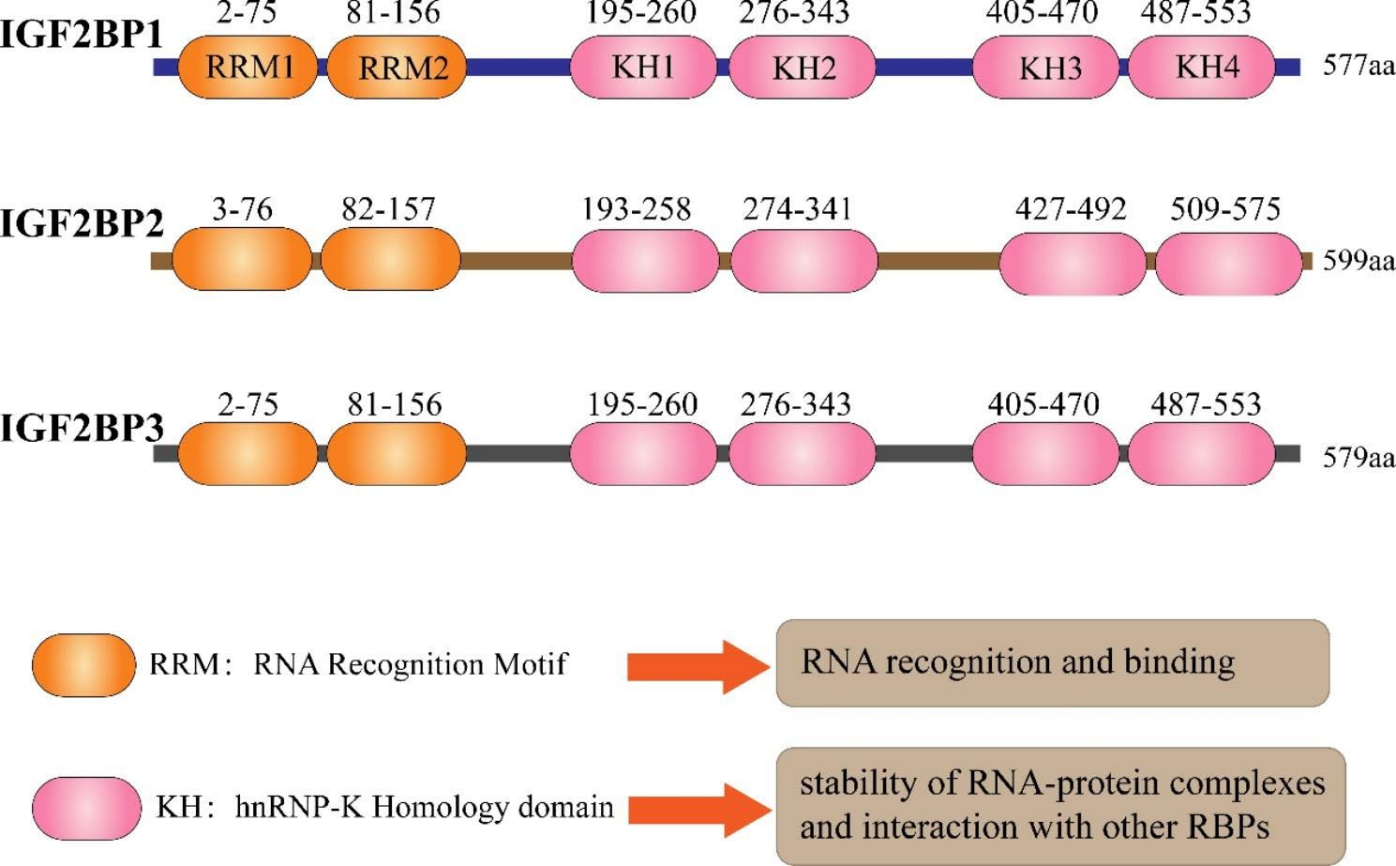
Features of human full-length IGF2BP protein family. Full-length IGF2BPs have a high identity in their domains, with two RRM (RNA recognition motif) and four KH (heterogeneous nuclear ribonucleoprotein K homology) domains. The RRM domain may mediate interactions with other RBPs (RNA-binding proteins) and the stability of RNA-protein complexes, and the KH domain is responsible for recognizing and binding RNA. The amino acid sequences of each domain in IGF2BPs are marked above them. Data from UniProt [130].

**Fig. 2:**
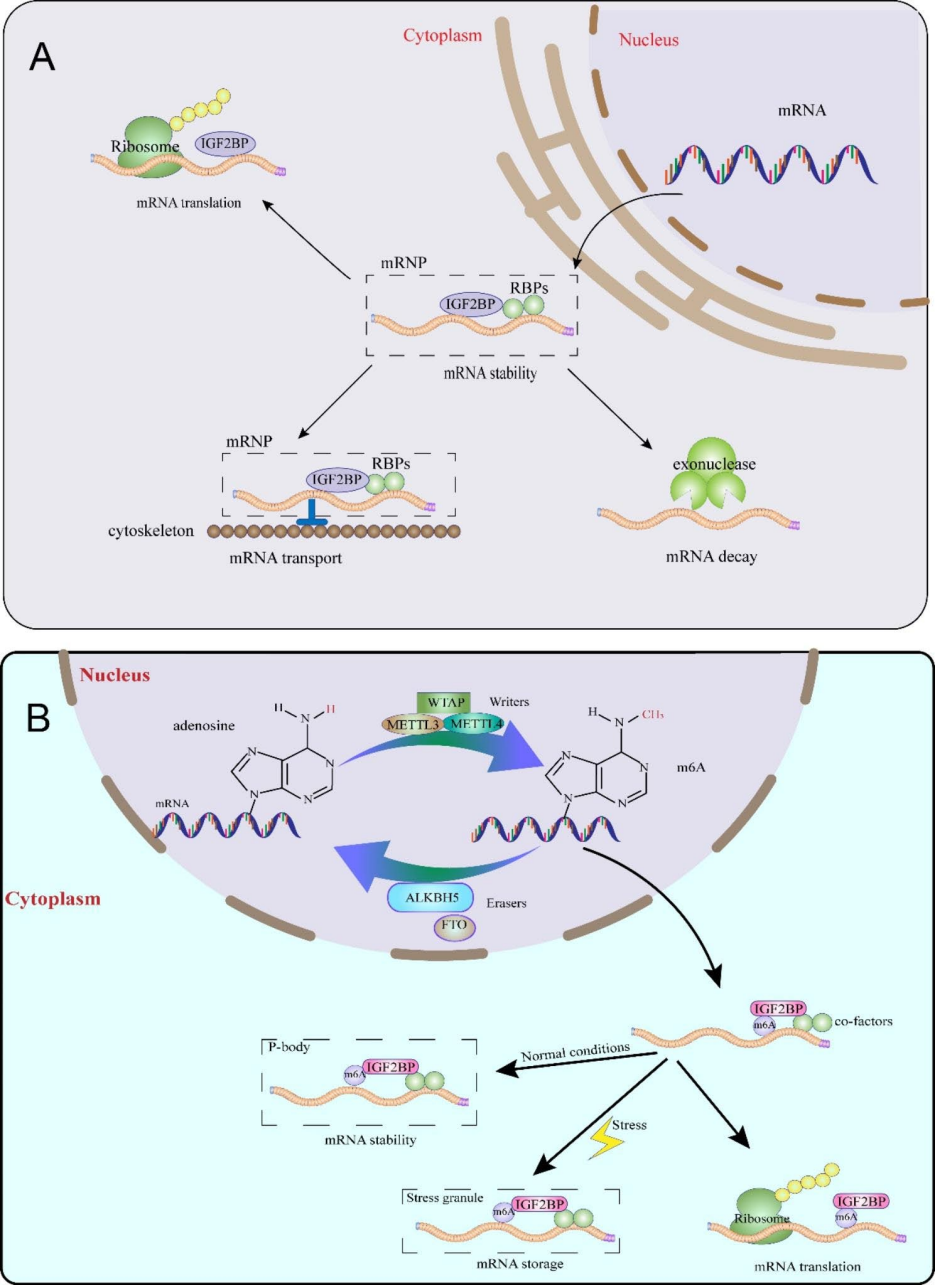
The post-transcriptional regulatory mechanisms of IGF2BPs. **A** IGF2BPs with other RBPs (RNA-binding proteins) regulate mRNA stability, transport, and translation by recruiting cytoplasmic transcripts into protective mRNPs (messenger ribonucleoprotein particles) including PBs (processing bodies) and SGs (stress granules). **B** IGF2BPs function as m^6^A readers to promote their stabilization and translation by selectively recognizing m^6^A-modified mRNAs under stress conditions in SGs or under normal conditions in PBs.

Insulin-like growth factor-2 mRNA-binding proteins (IGF2BPs, also known as IMPs or VICKZs), including IGF2BP1, IGF2BP2, and IGF2BP3, are a highly conserved family of RNA-binding proteins (RBPs) identified in humans since the late 20th century [1, 2]. They are named IGF2BPs due to their initial target, insulin-like growth factor II leader 3 mRNA [1]. In mammals, the three paralogues exhibit significant similarity in mass and structure, with molecular weight ranging from 58 to 66 kDa and amino acid sequence identities exceeding 56% [2]. This sequence conservation is most prominent in their domains, contributing to functional similarities (Fig. 1). IGF2BP proteins consist of two RNA recognition motif (RRM) domains (RRM-1 and RRM-2) and four heterogeneous nuclear ribonucleoprotein (hnRNP) K homology (KH) domains (KH-1 to KH-4) [1]. The C-terminal KH domains are responsible for recognizing and binding RNAs, while the N-terminal RRM domains likely contribute to the stability of RNA-protein complexes and interactions with other RBPs [3, 4]. With these domains, the three IGF2BP paralogues together target thousands of RNAs, of which approximately 92% are coding RNAs, with 55–70% co-targets. They play an essential role in mRNA processes such as transport, localization, stability, and translation [2, 5, 6]. Mechanically, IGF2BPs recruit their target transcripts to cytoplasmic messenger ribonucleoprotein particles (mRNPs) which subsequently condense into non-membrane-enclosed RNA granules, including processing bodies (PBs) and stress granules (SGs), thereby preventing mRNA decay and regulating translation-related events (Fig. 2A) [2, 7].

Additionally, IGF2BPs participate in various physiological and pathological processes throughout human life, from embryonic development to death, by controlling the expression of multitudinous genes. During embryogenesis, IGF2BPs are highly expressed and closely associated with cell differentiation and organ development [8]. Physiologically, all IGF2BPs, especially IGF2BP2, play a vital role in nervous system development and activity, as well as cell energy metabolism, including lipid metabolism, glycolysis, and aerobic respiration [8]. In contrast, IGF2BP1 and IGF2BP3 expression levels are negligible in adult tissues except for certain organs, while IGF2BP2 expression remains widespread [9]. However, gene expression analyses from Gene Expression Profiling Interactive Analysis (GEPIA) 2 (http://gepia2.cancer-pku.cn) [10] reveal significant re-expression of IGF2BP genes in most human cancer types. Moreover, mounting evidence suggests that aberrant expression of IGF2BPs contributes to various human tumors and participates in numerous malignant behaviors, including cell proliferation, invasion, stemness, apoptosis resistance, chemosensitivity, and immune escape. These effects are achieved by mediating several oncogene transcripts, such as KRAS, MYC, PTEN, and MDR1 [2, 8]. Consequently, IGF2BPs possess significant cancer-promoting properties, with IGF2BP1 and IGF2BP3 abnormally re-expressed in malignancies considered *bona fide* oncofetal proteins. IGF2BPs have regained widespread attention since their identification as a family of reader proteins that control RNA fate in an m^6^A-dependent manner [5]. Here, we mainly focus on recent advances in IGF2BPs’ roles as reader proteins and review the therapeutic potential of targeting IGF2BPs in cancers.

### RNA m^6^A modification and IGF2BPs

Epigenetics is the study of heritable changes in gene expression or cellular phenotype resulting from factors such as lifestyle and environment, rather than alterations of DNA sequence [11]. This field includes DNA methylation, RNA modification, histone modification, histone variants, non-coding RNA (ncRNA), and more [12]. Epitranscriptomics refers to epigenetics in RNA, encompassing internal chemical modifications such as 7-methylguanosine (m^7^G), m^1^G, m^6^A, m^1^A, 5-methylcytosine (m^5^C), m^3^C, and pseudouridine (Ψ), which plays a critical role in post-transcriptional control [13]. Nevertheless, m^6^A, the methylation at the nitrogen-6 position of adenosine, is the best-studied RNA modification since the 1970s. It has been identified as the most widespread and dynamic modification in both messenger RNA (mRNA) and ncRNA in eukaryotes, including mammals [14, 15]. Although m^6^A modifications are ubiquitous in mRNAs, transcriptome-wide m^6^A mapping has uncovered that m^6^A sites predominantly cluster near stop codons and within 3’ untranslated regions (3’UTRs), situated in a specific consensus motif RR (m^6^A) CH ( R = G/A; H = U/A/C) [16, 17].

As an epigenetic regulatory mechanism, m^6^A participates in diverse biological processes in most organisms through controlling gene expression and cell fate at the post-transcriptional level. These processes include spermatogenesis, cell self-renewal and differentiation, embryonic development, immunoregulation, and stress responses [18, 19]. The whole-transcriptome m^6^A profile of human fetal tissue shows a positive correlation between m^6^A methylation and gene expression homeostasis [20]. Disruption of m^6^A homeostasis can lead to various human diseases, such as psychiatric disorders, osteoporosis, obesity, type 2 diabetes, viral infection, and cancer [21].

This homeostasis is maintained by its primary regulators, including methyltransferases, demethylases, and effector proteins, which are also metaphorically referred to as writers, erasers, and readers, respectively [22, 23]. Methyltransferases are multi-subunit complexes comprising methyltransferase-like 3/14 (METTL3/14), Wilms tumor 1-associated protein (WTAP), RNA binding motif protein 15/15B (RBM15/15B), zinc finger CCCH-type containing 13 (ZC3H13), Vir like m^6^A methyltransferase associated (VIRMA), and Cbl proto-oncogene like 1 (CBLL1). These complexes recognize RNA targets and catalyze m^6^A methylation. However, only METTL3 functions as the catalytic subunit, with METTL3/14 considered core subunits [24, 25]. Notably, a recent study reported another m^6^A methyltransferase, METTL16, which can attach m^6^A to the 3’UTR of MAT2A to regulate its RNA splicing efficiency, further adjusting the homeostasis of S-adenosylmethionine (SAM), an influential methyl donor for m^6^A [26]. In contrast, N^6^-methylated adenosine in RNA can be reversed by demethylases, including AlkB homolog 5 (ALKBH5) and fat mass and obesity-associated protein (FTO), with the former playing a primary role. Writers and erasers are localized in the nucleus, where m^6^A is installed or removed in a co-transcriptional manner [18, 22]. Once the m^6^A modification in RNA is complete, effector proteins play an essential role in m^6^A-mediated RNA metabolism, including RNA splicing, translocation, stability, decay, and translation, through the selective recognition and binding of m^6^A sites (Table 1) [18, 23, 24]. In general, writers and erasers jointly maintain m^6^A homeostasis and confer reversible and dynamic properties to m^6^A methylation, while readers play a key role in determining the fate of m^6^A-modified RNAs.

**Table 1:** The roles of m^6^A readers on RNA metabolism

Reader	Function	Reference
IGF2BP1/2/3	promotes mRNA stability and translation	[5]
YTHDC1	promotes mRNA splicing and nuclear export	[27, 28]
YTHDC2	promotes mRNA translation initiation and instability	[29]
YTHDF1	enhances mRNA translation efficiency	[30]
YTHDF2	facilitates mRNA degradation	[31]
YTHDF3	affects mRNA translation and degradation through synergy with YTHDF1 and YTHDF2	[32]
eIF3	promotes mRNA cap-independent translation	[33]
METTL3	facilitates mRNA translation	[34]
FMR1	facilitates mRNA nuclear export, stability; competes with YTHDF1 for binding m^6^A sites to suppress translation	[35–37]
HNRNPC/G	mediates mRNA alternative splicing	[38, 39]
HNRNPA2B1	mediates primary microRNA processing and alternative splicing	[40]

Abbreviations: IGF2BP1/2/3, insulin-like growth factor-2 mRNA-binding protein 1/2/3; YTHDC1/2, YTH domain-containing protein 1/2; YTHDF1/2/3, YTH domain-containing family protein 1/2/3; eIF3, eukaryotic translation initiation factor 3; METTL3, methyltransferase-like 3; FMR1, fragile X messenger ribonucleoprotein 1; HNRNPC/G, heterogeneous nuclear ribonucleoprotein C/G; HNRNPA2B1, heterogeneous nuclear ribonucleoprotein A2B1

IGF2BPs, as a family of readers, preferentially recognize and selectively bind m^6^A-modified mRNAs to promote their stabilization and translation in an m^6^A-dependent manner under stress conditions in SGs or under normal conditions in PBs (Fig. 2B) [5]. The effect of IGF2BPs on target stability is likely exerted through recruiting co-factors such as ELAV-like RNA-binding protein 1 (ELAVL1; also known as HuR), matrin 3 (MATR3), and polyadenylate-binding protein 1 (PABPC1) [5]. Furthermore, numerous studies have shown that IGF2BPs play a carcinogenic role by stabilizing m^6^A-modified ncRNAs. The KH domains in IGF2BPs, particularly the KH3–4 di-domain, are confirmed to be responsible for m^6^A recognition and binding [5], which is consistent with the aforementioned KH functionality and has been corroborated in many subsequent studies. Remarkably, more than 80% of the thousands of IGF2BP targets contain at least one m^6^A-enriched region, and these regions spatially align with global m^6^A distribution [5], suggesting the significant role of IGF2BPs as reader proteins in the post-transcriptional regulation of m^6^A-methylated RNAs. However, the exact molecular mechanisms and functions of IGF2BPs in human cancers remain poorly understood.

### IGF2BPs function as m^6^A readers in human cancer

Since 2018, increasing evidence has demonstrated that dysregulated IGF2BPs play vital roles as m^6^A effector proteins in various malignancies, as summarized in Table 2.

**Table 2:** The roles of m^6^A reader IGF2BPs across cancer types

Cancer type	Reader	Writer/Eraser	Target	m^6^A site	RNA outcome	Cellular phenotypes	Ref
Acute myelocytic leukemia	IGF2BP2	METTL3/14	MYC, GPT2, SLC1A5	–	Stability, translation	Glutamine metabolism, stem cell self-renewal	[83]
	IGF2BP2	–	PRMT6	3’UTR	Stability	Stem cell maintenance	[53]
	IGF2BP3	–	RCC2	CDS region	Stability	Proliferation, apoptosis resistance	[65]
T cell acute lymphoblastic leukemia	IGF2BP2	METTL3	NOTCH1	–	Stability	Survival, chemo-resistance	[70]
Nasopharyngeal carcinoma	IGF2BP2	WTAP	lncRNA DIAPH1-AS1	1–281 nt	Stability	Proliferation, metastasis	[44]
	IGF2BP3	–	KPNA2	3’UTR	Stability	Proliferation, metastasis	[64]
Hypopharyngeal squamous cell carcinoma	IGF2BP2	ALKBH5	NFE2L2	3’UTR	Stability	Ferroptosis resistance	[54]
Laryngeal squamous cell carcinoma	IGF2BP3	RBM15	TMBIM6	3’UTR	Stability	Survival, metastasis	[60]
Papillary thyroid cancer	IGF2BP2	FTO	APOE	–	Stability	Glycolysis, proliferation	[82]
Breast cancer	IGF2BP3	METTL3	PD-L1	CDS region	Stability	Immune escape	[89]
Lung adenocarcinoma	IGF2BP1	METTL3/14	SRF	3’UTR	Stability	Growth, invasion	[41]
Gastric cancer	IGF2BP3	METTL3	HDGF	exon	Stability	Glycolysis, angiogenesis, proliferation, metastasis	[85]
	IGF2BP3	ALKBH5	PKMYT1	CDS region	Stability	Invasion, migration	[63]
	IGF2BP3	–	HIF1A	CDS region	Stability	Hypoxia-induced migration and angiogenesis	[86]
Hepatocellular carcinoma	IGF2BP1/2/3	METTL3/14	MYC	CRD	Stability, translation	Proliferation, metastasis	[5]
	IGF2BP1	METTL3/14	SRF	3’UTR	Stability	Growth, invasion	[41]
	IGF2BP1/3	METTL3	lnc-CTHCC	–	Stability	Proliferation, invasion	[58]
	IGF2BP2	METTL14	lncRNA ARHGAP5-AS1	928 nt	Stability	Proliferation, metastasis	[50]
	IGF2BP2	–	E2F3, E2F6	–	Stability	Stem cell self-renewal, metastasis	[51]
	IGF2BP3	METTL3/ALKBH5	PDK4	5’UTR	Stability	Glycolysis, proliferation	[72]
Pancreatic cancer	IGF2BP2	–	lncRNA DANCR	664 nt	Stability	Proliferation, stemness-like property	[43]
	IGF2BP2	METTL3	PLK1	3’UTR	Stability	Radio-resistance	[52]
Colorectal cancer	IGF2BP2	METTL3	SOX2	CDS region	Stability	Stemness maintenance, invasion, resistance to oxaliplatin	[69]
	IGF2BP2	METTL3	HK2; SLC2A1	5′/3’UTR; 3’UTR	Stability	Glycolysis, proliferation	[79]
	IGF2BP2	FTO	MTA1	CDS region	Stability	Metastasis	[45]
	IGF2BP2	METTL3	HMGA1	3’UTR	Stability	EMT, proliferation, metastasis	[46]
	IGF2BP2	METTL3	EphA2	3’UTR	Stability	Vasculogenic mimicry formation, proliferation, metastasis	[88]
	IGF2BP2	–	lncRNA ZFAS1	843 nt	Stability	Glycolysis, proliferation, apoptosis resistance, invasion	[80]
	IGF2BP2	–	MSX1, JARID2	–	Stability	Proliferation, apoptosis resistance, migration	[49]
	IGF2BP3	–	CCND1	CDS	Stability	Cell cycle regulation, proliferation	[56]
	IGF2BP3	–	VEGF	+ 2238 from the starting codon	Stability	Angiogenesis	[56]
	IGF2BP3	METTL3	VEGFA	3’UTR	Stability	Vasculogenic mimicry formation, proliferation, metastasis	[88]
	IGF2BP3	–	ABCB1	CDS	Stability	Resistance to ABCB1 substrates	[73]
	IGF2BP3	METTL3	SLC2A1	3’UTR	Stability	Glycolysis, proliferation	[79]
Renal cell carcinoma	IGF2BP1/2/3	WTAP	S1PR3	near stop codon	Stability	Migration, proliferation	[67]
	IGF2BP2	METLL3	ZNF677	CDS	Stability	Proliferation and apoptosis resistance	[48]
	IGF2BP2	–	PPAT	–	Stability	Proliferation	[55]
Clear cell renal cell carcinoma	IGF2BP2	FTO	SIK2	CDS	Stability	Autophagy regulation	[84]
	IGF2BP3	METTL3/14	CDK4	5’UTR	Stability	G1–S transition, proliferation, palbociclib resistance	[61]
	IGF2BP3	METTL3/14	FN1; COL6A1; LAMA5	the exon 20; the exon 15 and 3’UTR; the exon 4	Stability	ECM deposition, metastasis	[61]
Prostate cancer	IGF2BP2	METTL3	lncRNA PCAT6	–	Stability	Proliferation, metastasis	[47]
Ovarian cancer	IGF2BP1	METTL3/14	SRF	3’UTR	Stability	Growth and invasion	[41]
Endometrial cancer	IGF2BP1	ALKBH5	PEG10	3’UTR	Stability	Cell cycle regulation, proliferation	[42]
Cervical cancer	IGF2BP1/2/3	METTL3/14	MYC	CRD	Stability, translation	Proliferation, metastasis	[5]
	IGF2BP1	ALKBH5	FAAP20, ATRX	–	Stability	ROX-induced DNA damage repair	[66]
	IGF2BP3	METTL3/ALKBH5	PDK4	5’UTR	Stability	Glycolysis, proliferation, resistance to doxorubicin	[72]
	IGF2BP3	–	lncRNA KCNMB2-AS1	–	Stability	Proliferation, apoptotic resistance	[59]
	IGF2BP3	METTL3	ACIN1	–	Stability	Proliferation, migration	[57]
Seminoma	IGF2BP1	METTL3	TFAP2C	–	Stability	Resistance to cisplatin	[68]
Osteosarcoma	IGF2BP2	METTL14	MN1	CDS	Stability and translation	Resistance to all-trans retinoic acid, stemness maintenance, proliferation, metastasis	[71]

#### IGF2BPs in cancer stemness, survival and metastasis

Fundamental characteristics of cancers include immortalization, resistance to cell death, invasion, and metastasis.

***IGF2BP1.*** Müller S et al. have reported that IGF2BP1 enhances cancer cell growth and invasiveness by increasing the expression of transcription factor SRF via m^6^A modification and obstructing microRNA-dependent decay of SRF mRNA [41]. In endometrial cancer, IGF2BP1 regulates cell cycle and promotes cell proliferation through employing the cofactor PABPC1 and stabilizing m^6^A-modified PEG10 mRNA [42].

***IGF2BP2.*** IGF2BP2 maintains stemness and proliferation of pancreatic cancer cells by stabilizing long noncoding RNA (lncRNA) DANCR in an m^6^A-dependent manner [43]. LncRNA DIAPH1-AS1 is stabilized by IGF2BP2 via m^6^A modification that is mediated by WTAP, accelerating the growth and metastasis of nasopharyngeal carcinoma [44]. Increased MTA1 regulating colorectal cancer metastasis relies on FTO/IGF2BP2 regulated m^6^A methylation [45]. The formation of LINC00460/IGF2BP2/DHX9 complex reinforces HMGA1 mRNA stability in an m^6^A-dependent manner, promoting epithelial-mesenchymal transition (EMT), tumor growth, and metastasis in colorectal cancer [46]. METTL3 and IGF2BP2 control m^6^A of lncRNA PCAT6 and keep its stability. PCAT6 in turn intensifies the stability effects of IGF2BP2 on IGF1R mRNA, further promoting prostate cancer growth and bone metastasis [47]. ZNF677, which plays a tumor suppressor role in renal cell carcinoma, can be stabilized by METLL3/IGF2BP2-regulated m^6^A modification at the post-transcription level [48]. IGF2BP2 stabilizes MSX1 and JARID2 transcripts in an m^6^A-dependent manner, regulating cell migration and survival in colorectal cancer [49]. METTL14/IGF2BP2 prevents m^6^A-modified lncRNA ARHGAP5-AS1 degradation, playing an important part in hepatocellular carcinoma growth and metastasis [50]. IGF2BP2 functions as the m^6^A effector to stabilize E2F6 and E2F3, activating the Wnt/β-catenin pathway and facilitating liver tumor initiating cells (TICs) self-renewal and metastasis [51]. METTL3/IGF2BP2 regulates m^6^A modification of PLK1 and increases its stability and expression, leading to radio-resistance in pancreatic adenocarcinoma [52]. IGF2BP2 improves PRMT6 expression for leukemia stem cell (LSC) maintenance and acute myeloid leukemia development via stabilizing its transcript in an m^6^A-dependent form [53]. NFE2L2, upregulated by ALKBH5/IGF2BP2-mediated m^6^A methylation after transcription, inhibits ferroptosis of hypopharyngeal squamous cell carcinoma [54]. SHMT2 improves the m^6^A abundance of PPAT mRNA by offering methyl donor SAM, allowing IGF2BP2 to enhance the stability and expression of PPAT, regulating cell cycle to promote proliferation in renal cell carcinoma [55].

***IGF2BP3.*** In colon cancer, IGF2BP3 boosts DNA replication and cell proliferation by binding to m^6^A-modified sites in CCND1 mRNA and preventing its degradation [56]. ACIN1 is stabilized through METTL3/IGF2BP3, boosting proliferation and migration of cervical cancer cells [57]. In hepatocellular carcinoma, lnc-CTHCC, stabilized by METTL3/IGF2BP1/IGF2BP3 via m^6^A modification, promotes tumor initiation and development by binding hnRNPK and activating YAP1 transcription [58]. LncRNA KCNMB2-AS1 that improves IGF2BP3 expression via sponging miR-130b-5p and miR-4294 is stabilized by IGF2BP3 in an m^6^A-mediated manner, synergistically accelerating cervical cancer cell growth [59]. TMBIM6, a downstream target of RBM15 and IGF2BP3, is involved in proliferation, invasion, migration, and apoptosis of laryngeal squamous cell carcinoma cells [60]. In clear cell renal cell carcinoma, the complex of IGF2BP3/lncRNA DMDRMR selectively interacts with their m^6^A-modified co-targets (including CDK4, COL6A1, LAMA5, and FN1) to promote cell proliferation [61]. In prostate cancer, hsa_circ_0003258 directly binds IGF2BP3 and enhances its function of stabilizing m^6^A-methylated HDAC4 transcript, contributing to EMT and metastasis by stimulating the MAPK signaling pathway [62]. In gastric cancer, IGF2BP3 physically binds and stabilizes PKMYT1 mRNA in an m^6^A-dependent manner, facilitating cell invasion and migration [63]. IGF2BP3 recognizes and reads m^6^A sites in KPNA2 mRNA to promote its stability and expression, activating tumor growth and metastasis in nasopharyngeal carcinoma [64]. In acute myeloid leukemia, IGF2BP3 is indispensable for leukemia cell survival by enhancing RCC2 expression through the interaction between IGF2BP3 and m^6^A sites in RCC2 mRNA [65].

***IGF2BPs.*** In hepatocellular carcinoma cells and HeLa cells, knockdown of each IGF2BP can hinder MYC expression and tumor proliferation, colony formation, migration, and invasion by suppressing MYC mRNA stability and translation in an m^6^A-dependent manner [5]. In HEK293T and HeLa cells, reactive oxygen species (ROS) signaling improves m^6^A abundance by weakening the demethylase activity of ALKBH5, which withstands ROX-induced DNA damage and apoptosis through stabilization of m^6^A–modified FAAP20 and ATRX mRNA mediated by IGF2BP proteins [66]. IGF2BPs facilitate cell proliferation and migration in renal cell cancer by stabilizing S1PR3 mRNA in an m^6^A-dependent manner and activating the PI3K/AKT pathway [67].

#### IGF2BPs in drug resistance

***IGF2BP1.*** In seminoma cells, cisplatin induces a significant elevation of METTL3 and m^6^A levels, and IGF2BP1 stabilizes TFAP2C gene in an m^6^A-dependent manner in response to the chemotherapy damage [68].

***IGF2BP2.*** In colorectal carcinoma, m^6^A modification of SOX2 is installed by METTL3 and recognized by IGF2BP2, resulting in upregulated SOX2 that strengthens cell stemness, invasion property, and resistance to oxaliplatin [69]. In T-cell acute lymphoblastic leukemia, IGF2BP2 impels cell chemoresistance and survival via directly binding and stabilizing m^6^A-modified NOTCH1 mRNA [70]. In addition, METTL14-mediated MN1 methylation raises the stability and translation of MN1 transcript through the IGF2BP2-dependent pathway, prompting all-trans-retinoic acid resistance and progression in osteosarcoma [71].

***IGF2BP3.*** In cervical and liver cancers, m^6^A in PDK4 transcript regulated by IGF2BP3 and METTL3 plays an important role in tumor growth and chemoresistance [72]. Overexpression of ABCB1 inducing multidrug resistance (MDR) desensitizes colon cancer cells to chemotherapy drugs with ABCB1 substrate specificity, resulting from enhanced stability of ABCB1 transcript via IGF2BP3 reading its m^6^A region [73]. In clear cell renal cell carcinoma, IGF2BP3 suppresses cancer sensitivity to CDK4/6 inhibitor palbociclib by stabilizing CDK4 mRNA in an m^6^A- dependent manner [61].

#### IGF2BPs in reprogramming of metabolism

The Warburg effect, a hallmark of cancer cells, describes their preference for obtaining energy through aerobic glycolysis instead of oxidative phosphorylation [74, 75]. Glutamine presents metabolic dysregulation in cancer and serves as a significant source of nitrogen and carbon for cancer development [76]. These processes support cancer progression directly or through enhancing crosstalks with the surrounding tumor microenvironment (TME), which includes immune cells, fibroblasts, endotheliocytes, and extracellular matrix [77]. Besides, autophagy, responsible for the degradation and recycling of dysfunctional organelles and proteins, plays dual roles in both promoting and suppressing cancer through adjusting cellular metabolism [78].

***IGF2BP2.*** HK2 and GLUT1, the key factors of glucose metabolism, undergo m^6^A modification by METTL3 and are individually stabilized by IGF2BP2 and IGF2BP2/3 at the post-transcription level, activating the Warburg effect to promote colorectal cancer progression[79]. In colorectal cancer, IGF2BP2 stabilizes lncRNA ZFAS1 in an m^6^A-dependent manner, which activates OLA1 to reinforce adenosine triphosphate (ATP) hydrolysis and aerobic glycolysis [80]. IGF2BP2 also can enhance colorectal cancer cell glycolysis through the downstream MYC gene [81]. In papillary thyroid cancer, low-level FTO results in m^6^A modification in APOE mRNA, which is then read and stabilized by IGF2BP2, activating glycolysis through IL-6/JAK2/STAT3 signaling [82]. In acute myeloid leukemia, IGF2BP2 enhances the mRNA stability and translation initiation of m^6^A-containing targets GPT2, SLC1A5, and MYC by employing PABPC1 and eukaryotic translation initiation factor (eIF) complexes eIF4A, respectively, facilitating glutamine uptake and metabolism for tumor cell stemness and development [83]. In clear cell renal cell carcinoma, SIK2 downregulation promotes tumor progression by inhibiting autophagy, depending on FTO/IGF2BP2-m^6^A axis-mediated SIK2 mRNA stabilization [84].

***IGF2BP3.*** IGF2BP3 can increase the expression of PDK4 by binding to the m^6^A site and enhancing its mRNA stability, thereby promoting glycolysis and ATP production in cervical and liver cancer cells [72]. In addition, IGF2BP3 and METTL3 improve the expression of transcription factor HDGF by directly recognizing and binding the m^6^A-modified HDGF, which transcriptionally accelerates the expression of GLUT4 and ENO2 to facilitate glycolysis in gastric cancer. This ultimately results in pro-tumorigenic activities, including cancer growth and liver metastasis [85].

#### IGF2BPs in tumor microenvironment

The TME comprises various cell types (including fibroblasts, endothelial cells, and immune cells) and noncellular components (including extracellular matrix, cytokines, and metabolites). The TME can be considered the “soil” in which cancer cells, the “seeds”, grow. The crosstalk between the two creates a complex ecosystem involved in multiple aspects of malignant development, including tumor growth, metastasis, drug resistance, angiogenesis, and immune escape.

***IGF2BP2/3.*** In clear cell renal cell carcinoma, IGF2BP3 promotes extracellular matrix (ECM) deposition, including COL6A1, LAMA5, and FN1, by stabilizing their m^6^A-methylated transcripts [61]. As previously discussed, HDGF expression is fostered via the METTL3/IGF2BP3-HDGF mRNA axis in an m^6^A-dependent manner in gastric cancer. Intriguingly, while nuclear HDGF involves in glycolysis, secretory HDGF in the TME promotes tumor angiogenesis [85]. Another different mechanism of angiogenesis in gastric cancer has been revealed: the disruption of IGF2BP3 weakens the stability of m^6^A-enriched HIF1A, consequently inhibiting hypoxia-induced cell migration and angiogenesis [86]. In colon cancer, IGF2BP3 expression promotes angiogenesis by targeting m^6^A-methylated VEGF mRNA [56]. Beyond traditional tumor angiogenesis, cancer cells can form channels resembling blood vessels, which are responsible for supplying blood, nutrients, and oxygen. This process is termed vasculogenic mimicry [87]. In colorectal cancer, IGF2BP2 and IGF2BP3 can stimulate vasculogenic mimicry formation by separately stabilizing the transcripts of EphA2 and VEGF via interaction with their m^6^A sites. This subsequently activates downstream PI3K/AKT/mTOR and MAPK/ERK1/2 signaling pathways, promoting cell proliferation, migration, and invasion [88]. Additionally, the METTL3/IGF2BP3 axis reinforces the immune evasion capacity of breast cancer cells in the TME through increasing the stability of downstream target PD-L1 mRNA and the expression of its transmembrane protein [89].

To sum up, IGF2BPs function as oncogenes promoting various aspects of tumor properties, including tumorigenesis, survival, metastasis, drug resistance, metabolic reprogramming, matrix deposition, angiogenesis, and immune evasion, by binding to m^6^A-containing RNAs and increasing their stability and/or translation. In addition to their role as RBPs, the identification of m^6^A readers means a deeper exploration of IGF2BPs. Further systematic studies are needed to fully elucidate the essence of IGF2BPs, including their effects on hallmarks across cancer types, alternative mechanisms, target selectivity, the roles of each domain, and more.

### The value of IGF2BPs in clinical prediction

As discussed above, IGF2BPs typically paly a pro-carcinogenic role in human malignancies and are involved in numerous aspects of tumor characteristics. Evidence has shown that IGF2BPs are often aberrantly overexpressed and could serve as biomarkers for diagnosis and prognosis across various cancer types.

Tumor-associated antigens (TAAs), such as carcinoma embryonic antigen (CEA), alpha-fetoprotein (AFP), and carbohydrate antigen 125 (CA125), are widely used in clinical tumor diagnosis. IGF2BPs, as embryonic proteins, have been found to be valuable for early screening and diagnosis of tumors. In colorectal cancer, IGF2BP1 and IGF2BP3 might act as potential biomarkers for screening high-risk groups and cancer patients [90]. In renal cell carcinoma, IGF2BP3 serves as an indicator of high risk for metastasis and informs systemic treatment decisions [91]. Yang et al. suggested that IGF2BP3 and IGF2BP2 could be considered specific genes of triple-negative breast cancer, the molecular subtype with the poorest prognosis, which would aid in more precise diagnosis and treatment for breast cancer patients [92]. Gong Y et al. reviewed IGF2BP3 as a competent molecular marker for diagnosis in a majority of malignancies [93].

While numerous studies have highlighted the potential of histological detection of IGF2BP proteins for tumor diagnosis, body fluid tests offer a more convenient and minimally invasive approach for patients. The detection of serum autoantibodies to IGF2BP1 in esophageal squamous cell carcinoma [94] and IGF2BP1/3 in colon cancer [95] contributes to identifying patients with cancer, and when combined with other TAAs, achieves greater sensitivity and specificity for diagnosis. Moreover, autoantibodies to IGF2BP2 were detected in the serum of some hepatocellular carcinoma patients but not in precancerous lesions like chronic hepatitis or liver cirrhosis hepatitis [96]. Zhang J et al. expanded autoantibody testing of IGF2BP2/3 to more cancers and believed that they could become valuable biomarkers for pan-cancer clinical applications [97]. Given the broad expression and oncogenic roles of IGF2BPs in cancers, their detection could be beneficial for the diagnosis of other tumors not mentioned above.

In addition, IGF2BPs have been suggested for tumor prognosis assessment. IGF2BP3 might be a pan-cancer oncogene, as its overexpression is associated with poor patient survival in various cancers, including kidney renal clear cell carcinoma, kidney renal papillary cell carcinoma, brain lower-grade glioma, and more [98]. Moreover, IGF2BP3 is upregulated and its high expression conveys tumor progression and worse prognosis in patients with lung adenocarcinoma [99], esophageal cancer [100], gastric cancer [63], pancreatic adenocarcinoma [101], papillary renal cell carcinoma [102], colon cancer [56], clear cell renal cell carcinoma [61], bladder cancer [103], prostate cancer [104], nasopharyngeal carcinoma [64], laryngeal squamous cell cancer [60], melanoma [105], glioma [106], acute myelocytic leukemia [65], among others. IGF2BP1 has been identified as an unfavorable prognostic indicator in tumors such as lung adenocarcinoma [107], endometrial cancer [42], gastric cancer [108], hepatocellular carcinoma, and serous ovarian cancer [41]. Upregulated IGF2BP2 is significantly correlated with weak prognosis, providing a hopeful predictor for pancreatic cancer [43], colorectal cancer [69, 80, 81], osteosarcoma [71], and lung adenocarcinoma [99].

The immune microenvironment and immunotherapy have become research hotspots, especially since the discovery of immune checkpoint therapy earned a Nobel Prize. The mechanism by which IGF2BP3 promotes PD-L1 expression has been stated above. Both proteins are highly expressed and positively correlated in breast cancer, especially in HER2-enriched and triple-negative breast cancer, the more aggressive subtypes [89]. IGF2BP3 also exerts a significant impact on PD-L1 expression in bladder cancer [109]. Besides, IGF2BPs have been found to be associated with immunomodulators and immune infiltration levels in diverse cancers [99, 101, 105, 107, 110–114]. Consequently, IGF2BPs may provide guidance for screening patients responsive to immunotherapy and could become potential targets for boosting immunotherapy response.

In conclusion, IGF2BPs show promise as broad-spectrum tumor markers for the diagnosis and prediction of tumors, and their performance improves when combined with other TAAs. However, IGF2BPs only provide diagnostic clues and lack tissue and organ specificity. Additionally, IGF2BP2 is expressed in multiple normal organs, which reduces tumor specificity. As a result, a baseline value must be established to evaluate normal or abnormal expression. Further research is needed to determine whether the expression of IGF2BPs is related to disease severity and tumor stage, and whether they can monitor treatment efficacy, recurrence, and metastasis.

### The therapeutic potential of targeting IGF2BPs

**Fig. 3:**
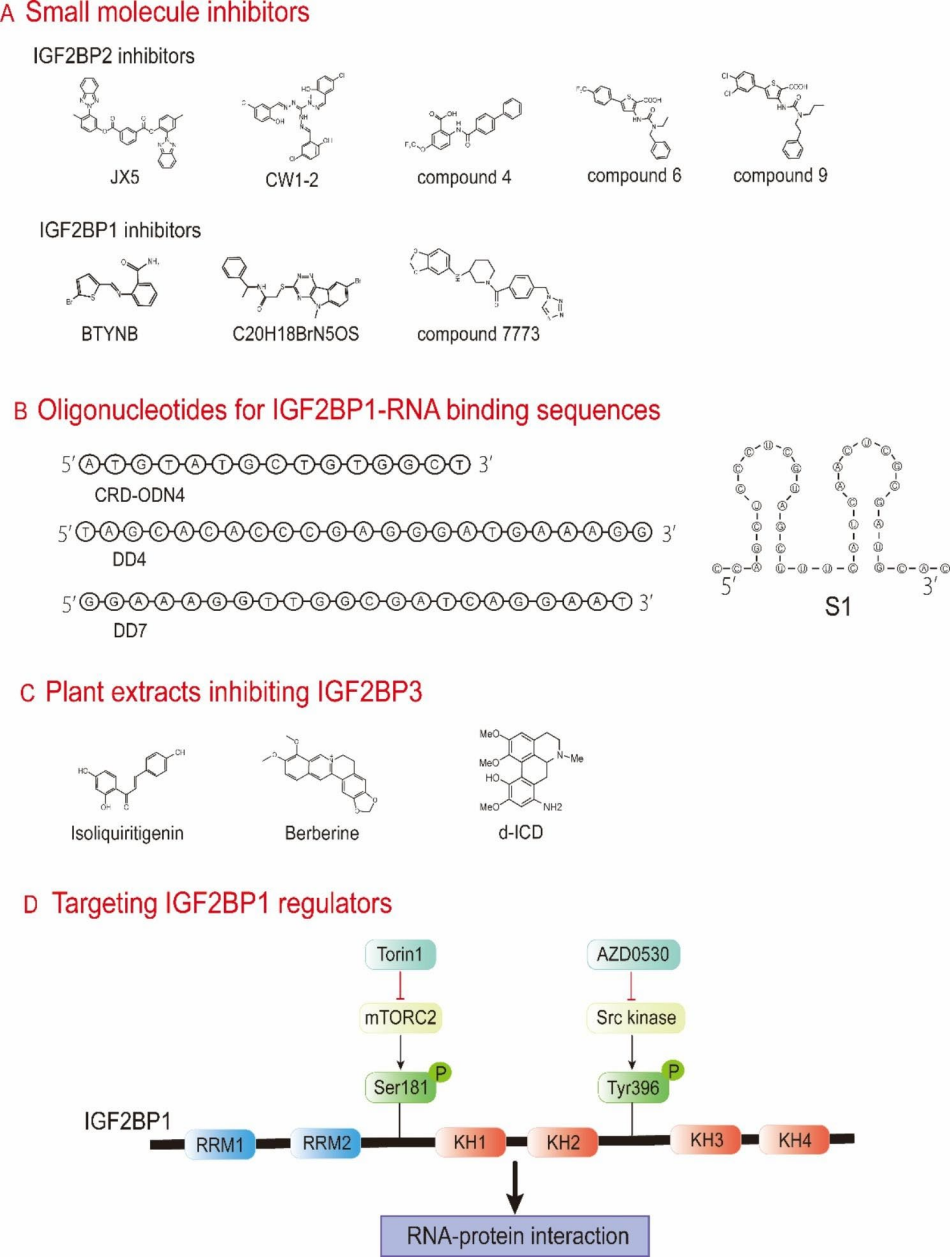
Potential therapeutic strategies of targeting IGF2BPs in cancer. **A** The structure of small molecule inhibitors for IGF2BP1/2. **B** Oligonucleotide sequences designed according to IGF2BP1-RNA binding sites. **C** Natural plant extracts that effectively inhibit IGF2BP3 expression. d-ICD: derivative of isocorydine. **D** Torin1 (mTORC2 kinase inhibitor) and AZD0530 (Src kinase inhibitor) impede IGF2BP1-RNA interaction by hindering IGF2BP1 phosphorylation.

The therapeutic potential of targeting IGF2BPs is gaining attention due to advances in understanding their molecular structures and carcinogenic mechanisms. We summarized all the current agents and strategies for targeting IGF2BPs (Fig. 3).

#### Small molecule inhibitors

Small molecule inhibitors have emerged as a promising strategy for cancer treatment, with many small molecule drugs already used in clinical and showing encouraging results. Recently, a specific small molecule inhibitor, JX5 (Kd value = 93.2 ± 3.9 µM), has been identified for IGF2BP2 through high-throughput virtual screening based on the three-dimensional structure of IGF2BP2 [70]. It inhibits IGF2BP2 activity by physically docking with its KH3-4 di-domains and has shown effectiveness in hampering T-cell acute lymphoblastic leukemia cell amplification and marrow/spleen infiltration with minimal toxicities [70]. Furthermore, IGF2BP2 inhibitors, CWI1-1 and CWI1-2, have also been identified, with CWI1-2 (IC50 = 203.1-781.6 nM in leukemia cell lines) demonstrating stronger anti-tumor effects on acute myelocytic leukemia *in vitro* and *in vivo* [83]. Importantly, CWI1-2 has shown synergistic effects when combined with conventional acute myelocytic leukemia chemotherapy [83]. Several compounds, belonging to either benzamidobenzoic acid or ureidothiophene, have been found to selectively restrain the interaction of IGF2BP2 with its target RNAs [115]. Among them, compound 4 (IC50 = 18.2–35.5 µM), compound 6 (IC50 = 42.9–52.6 µM), and compound 9 (IC50 = 24.9–39.8 µM) demonstrate higher activity and effectively kill IGF2BP2-containing colorectal cancer and hepatocellular carcinoma cells *in vitro* and *in vivo* [115]. Small molecule allosteric inhibitor BTYNB (IC50 = 2.3 µM in ES-2, = 3.6 µM in IGROV-1, = 4.5 µM in SK-MEL2) has been identified to hinder the proliferation of IGF2BP1-overexpressing ovarian cancer and melanoma cells by altering the functional site of IGF2BP1 and decreasing the expression of downstream oncogenes, including MYC, BTRC, and eEF2 [116]. Intriguingly, BTYNB has been found to reduce both IGF2BP1 and IGF2BP2 protein levels [83]. The compound 2-[(8-bromo-5-methyl-5 H-[1,2,4]triazino[5,6-b]indol-3-yl)thio]-N-(1-phenylethyl)acetamide (C_20_H_18_BrN_5_OS, ChemBridge ID: 6896009) has been regarded as a lead IGF2BP1 inhibitor to obstruct the binding of IGF2BP1 to MYC mRNA and can obviously restrain IGF2BP1-containing ovarian cancer cell proliferation [117]. In non-small-cell lung carcinoma and ovarian clear cell carcinoma, another IGF2BP1 small molecule inhibitor compound 7773 competitively binds to a hydrophobic surface around KH3-4 domains and blocks their binding activity to target transcripts, which inhibits cell migration and growth without any toxicity [118].

Unfortunately, no specific small molecule inhibitors have been discovered for IGF2BP3 yet. However, given the high similarity among IGF2BP paralogues, especially in their structural domains, it is possible that some small molecule inhibitors targeting one IGF2BP could also target the other two proteins, potentially having therapeutic effects on the tumors they drive. For example, BTYNB has already been verified to target both IGF2BP1 and IGF2BP2, inhibiting leukemia initiation and development [115]. Further studies are warranted to explore this possibility and to develop more specific inhibitors targeting IGF2BPs for cancer therapy.

#### Oligonucleotides

Oligonucleotides, including antisense oligonucleotides (ASOs), small interfering RNAs (siRNAs), microRNAs (miRNAs), and aptamers, hold promise as cancer therapeutics by regulating gene expression and product function [119].

IGF2BPs interact with specific RNA sites to promote gene expression, and ASOs can be designed to target these specific sequences and disrupt these interactions. For example, ASOs CRD-ODN4 [120] and DD4/DD7 [121] have been designed to target the specific sequences recognized by IGF2BP1 on MYC and CD44 mRNA, respectively, and have been shown to efficiently reduce intracellular MYC and CD44 levels.

In addition to inhibiting gene expression through complementary base pairing, oligonucleotides can also interact with and block proteins through their three-dimensional secondary structures. The structured oligonucleotide S1, containing two distinct stem loops, has been shown to target IGF2BP1 domains and interferes with IGF2BP1-GLI1 mRNA interaction [122]. Moreover, 2’-O-methyl derivatives of these oligonucleotides can lower mRNA levels of corresponding oncogenes in diverse cancer cells [120–122].

siRNAs are principal components of the RNA-induced silencing complex (RISC), which mediates gene silencing of complementary target transcripts. siRNA drugs have been approved by the Food and Drug Administration (FDA) for the treatment of clinical diseases [119]. There is substantial evidence that siRNA can be used to specifically knock down IGF2BPs in experiments and effectively suppress IGF2BP-mediated malignant phenotypes *in vitro* and *in vivo*.

However, there are significant challenges associated with oligonucleotide therapeutics, including the development of efficient delivery systems, the potential for off-target effects, and unknown toxicities [119]. Continued research is needed to address these challenges and optimize oligonucleotide-based therapies targeting IGF2BPs for cancer treatment.

#### Plant extracts

Plant extracts and their derivatives have been widely applied to clinical antitumor strategies, with well-known examples including vinca alkaloids, camptothecins, taxanes, anthracyclines, and podophyllotoxin [123]. Some plant extracts, such as isoliquiritigenin [124], berberine [125], and an isocorydine derivative (d-ICD) [126] have been shown to decrease IGF2BP3 expression, subsequently inhibiting the malignant behavior of cancers.

#### Targeting regulators of IGF2BPs

Targeting the dysregulated upstream regulators of IGF2BPs, including their transcription factors, epigenetic ncRNAs, post-translational modification, and E3 ligase-mediated ubiquitination, is another potential treatment strategy for IGF2BP-driven cancer. For example, IGF2BP1 phosphorylation at Ser181 and Tyr396, catalyzed by mTORC2 and Src kinase, respectively, is involved in the interaction and post-transcriptional regulation of target transcripts such as MYC [127–129]. And mTORC2 kinase inhibitor Torin1 and Src kinase inhibitor AZD0530 have been identified to synergistically inhibit the growth of IGF2BP1-expressing cancer cells *in vitro* and *in vivo* by disrupting IGF2BP1 phosphorylation [129].

Given the significant tumor-promoting abilities of IGF2BPs in various malignancies, targeting dysregulated IGF2BPs is an appealing approach for cancer treatment. However, the clinical application of agents targeting IGF2BPs is still in its early stages. Several challenges need to be addressed, including improving the understanding of IGF2BPs’ structures and carcinogenic mechanisms, selecting suitable drug candidates, conducting credible preclinical studies, and performing rigorous clinical trials.

## Conclusion

As the most widespread and energetic RNA internal modification, m^6^A is involved in almost every aspect of RNA metabolism to control gene expression and cell phenotype. The imbalance of m^6^A modification caused by maladjusted m^6^A regulators, including IGF2BPs, has become a driver of tumor initiation and progression. Since the identification of IGF2BPs as a family of reader proteins, substantial studies that implicate the role of IGF2BPs in diverse malignancies have emerged, providing novel insights into the carcinogenic mechanisms of IGF2BPs. IGF2BPs play a crucial role in m^6^A-mediated post-transcriptional regulation. They preferentially recognize and bind m^6^A-modified target RNAs to promote their stabilization and translation in an m^6^A-dependent manner. IGF2BPs also function in RNA processes independently of m^6^A modification. However, the mechanism by which IGF2BPs affect RNA metabolism remains unclear, representing further research direction. Consequently, dysregulated IGF2BPs accumulate oncogenic products to promote various malignant phenotypes, including proliferation, resistance to cell death, metastasis, drug resistance, metabolism reprogramming, angiogenesis, and immune escape.

IGF2BPs, particularly IGF2BP1 and IGF2BP3, are oncofetal proteins and exhibit significantly up-regulated expression and prognostic correlation in various human cancers. Furthermore, IGF2BPs could serve as broad-spectrum tumor markers for early screening and prognosis evaluation. Combining these markers with others in specific cancers may enhance the accuracy of diagnosis, outcome prediction, and treatment guidance.

Understanding the underlying mechanisms of IGF2BPs in tumorigenic processes and their molecular structures is essential for developing therapeutic strategies. Small molecule inhibitors targeting IGF2BP1 and IGF2BP2 have been developed based on their KH3-4 structural domain, the core region that recognizes and binds RNA. However, further *in vivo* efficacy and toxicity studies are highly warranted. In addition, targeting IGF2BP1 and IGF2BP3 could be more specific for tumors and safer for patients, given their absence in normal tissues and high levels in many cancers.

In summary, IGF2BPs play a wide range of roles in cancer biology through post-transcriptional regulation of gene expression in both m^6^A-dependent and m^6^A-independent forms. They have shown potential as new biomarkers for tumor early screening, diagnosis, and prognosis. Current cancer therapies targeting IGF2BPs are still in their infancy. Further research into the molecular structure of IGF2BPs and their regulatory mechanisms in cancer is necessary to develop novel effective cancer therapies.

## Data Availability

Not applicable.

## Abbreviations

m^6^A: N^6^-methyladenosine.
IGF2BPs: Insulin-like growth factor-2 mRNA-binding proteins
RBPs: RNA-binding proteins
RRM: RNA recognition motif
hnRNP: Heterogeneous nuclear ribonucleoprotein
KH: Heterogeneous nuclear ribonucleoprotein K homology domain
mRNPs: Messenger ribonucleoprotein particles
PBs: Processing bodies
SGs: Stress granules
ncRNA: Non-coding RNA
m^7^G: 7-methylguanosine
m^5^C: 5-methylcytosine
Ψ: Pseudouridine
mRNA: Messenger RNA
UTRs: Untranslated regions
METTL3/14: Methyltransferase-like 3/14
WTAP: Wilms tumor 1 associated protein
RBM15/15B: RNA binding motif protein 15/15B
ZC3H13: Zinc finger CCCH-type containing 13
VIRMA: Vir like m^6^A methyltransferase associated
CBLL1: Cbl proto-oncogene like 1
SAM: S-adenosylmethionine
ALKBH5: AlkB homolog 5
FTO: Fat mass and obesity-associated protein
YTHDC1/2: YTH domain-containing protein 1/2
YTHDF1/2/3: YTH domain-containing family protein 1/2/3
eIF3: Eukaryotic translation initiation factor 3
FMR1: Fragile X messenger ribonucleoprotein 1
HNRNPC/G/A2B1: Heterogeneous nuclear ribonucleoprotein C/G/A2B1
ELAVL1: ELAV-like RNA-binding protein 1
MATR3: Matrin 3
PABPC1: Polyadenylate-binding protein 1
lncRNA: Long noncoding RNA
ROS: Reactive oxygen species
TME: Tumor microenvironment
ATP: Adenosine triphosphate
ECM: Extracellular matrix
TAA: Tumor-associated antigen
ASO: Antisense oligonucleotide
siRNA: Small interfering RNA
CDS: Coding sequence region
