# Correction: Oncofetal protein IGF2BPs in human cancer: functions, mechanisms and therapeutic potential

**DOI:** 10.1186/s40364-023-00512-6

**Published:** 2023-07-21

**Authors:** Tian-Yu Zhu, Lian-Lian Hong, Zhi-Qiang Ling

**Affiliations:** 1 Zhejiang Cancer Hospital, Hangzhou, 310022 Zhejiang China; 2 Hangzhou Institute of Medicine (HIM), Chinese Academy of Sciences, Hangzhou, 310018 Zhejiang China; 3 The Second School of Clinical Medicine, Wenzhou Medical University, No.109 Xueyuan West Road, Wenzhou, Zhejiang, 325027 China; 4 Jinhua People’s Hospital, No.267 Danxi East Road, Jinhua, Zhejiang, 321000 China

**Biomarker Research (2023) 11:62**

10.1186/s40364-023-00499-0

The original article [1] mistakenly omitted the following Funding information:

**Funding:**

This work was funded by National Natural Science Foundation of China (81,972,908, 32,271,238), National Health Commission Science Research Fund-Zhejiang Provincial Health Key Science and Technology Plan Project (WKJ-ZJ-2117), Leading Talents in Scientific and Technological Innovation from Zhejiang Provincial Ten Thousand Talents Plan (Zhejiang Provincial CPC Committee Talents [2019]-3), Zhejiang Province Health Leader Talent (Zjwjw2021-40), and Major Training Personnel from Zhejiang Provincial Program for Training and Development Project for 151 Talents (Zjhrss2014-150).
